# Correction of complex nonlinear signal response from a pixel array detector

**DOI:** 10.1107/S1600577515005536

**Published:** 2015-04-22

**Authors:** Tim Brandt van Driel, Sven Herrmann, Gabriella Carini, Martin Meedom Nielsen, Henrik Till Lemke

**Affiliations:** a Linac Coherent Light Source, SLAC National Accelerator Laboratory, 2575 Sand Hill Road, Menlo Park, CA 94025, USA; b Department of Physics, Technical University of Denmark, 2800 Kgs Lyngby, Denmark

**Keywords:** FEL, detector nonlinearity, diffuse scattering

## Abstract

The pulsed free-electron laser light sources represent a new challenge to photon area detectors due to the intrinsic spontaneous X-ray photon generation process that makes single-pulse detection necessary. Intensity fluctuations up to 100% between individual pulses lead to high linearity requirements in order to distinguish small signal changes. In real detectors, signal distortions as a function of the intensity distribution on the entire detector can occur. Here a robust method to correct this nonlinear response in an area detector is presented for the case of exposures to similar signals. The method is tested for the case of diffuse scattering from liquids where relevant sub-1% signal changes appear on the same order as artifacts induced by the detector electronics.

## Abstract 2

A procedure to correct signal-dependent nonlinear signal distortions in an area detector is introduced and tested on diffuse scattering signals measured at a free-electron laser.

## 1. Introduction   

Free-electron lasers (FELs) represent a new type of X-ray light source with different properties and requirements than synchrotron sources. These pose a challenge to experimenters, predominantly from the synchrotron community where the beam parameters are significantly more stable. The self-amplified spontaneous emission (SASE) process creates spontaneously emitted, very intense and transverse coherent X-ray pulses (∼1 mJ per pulse), which fluctuate in properties such as pointing, intensity (up to 100%), photon energy and arrival time. In order to sort and select pulses for these properties, single-pulse detection is essential (≤120 Hz pulse rate for presently operating facilities). For area detectors, this has led to special developments of direct detection and fast-readout integrating detectors (Henrich *et al.*, 2011 ▶; Weidenspointner *et al.*, 2011 ▶; Koch *et al.*, 2013 ▶; Mozzanica *et al.*, 2014 ▶). At the Linac Coherent Light Source (LCLS), the Cornell–SLAC Pixel Array Detector (CSPAD) has been developed and deployed. This 14-bit hybrid pixel detector, based on ASICs with 194 × 185 110 µm × 110 µm pixels, reads out at 120 Hz (Philipp *et al.*, 2010 ▶, 2011 ▶; Herrmann *et al.*, 2013 ▶; Hart *et al.*, 2012 ▶; Carini *et al.*, 2014 ▶; Blaj *et al.*, 2015 ▶). The detector has down to single photon sensitivity (at 8 keV and at the few photons per pixel limit) and can be combined to a 170 mm × 170 mm 2.3 megapixel camera for applications in crystallography or diffuse scattering (Boutet *et al.*, 2012 ▶; Trigo *et al.*, 2013 ▶; Sellberg *et al.*, 2014 ▶; Arnlund *et al.*, 2014 ▶).

While the first prototypes of the CSPAD were characterized by a significant non-uniformity of the ASIC, further iterations led to a much more uniform response and improved noise performance of the detector (Herrmann *et al.*, 2014 ▶). Another artifact exacerbated by the fast readout is pixel crosstalk, which depends highly on the exposure of the detector (Herrmann *et al.*, 2013 ▶). In effect, the pixel sensitivity depends both on the intensity and spatial distribution of the exposure. For strong diffuse signals, *i.e.* from liquid scattering, correction for such effects is very challenging, because the entire detector module is flooded with signal and normalization to local reference data in the detector pattern is not possible. Especially in the case of ultrafast pump/probe experiments, one of the major applications of LCLS, the signal changes after excitation with an optical light pulse are often below 1%. Nonlinear effects, for example due to crosstalk, here often outweigh the physical signal significantly and make it impossible to measure any sensible signal without special data treatment. Efficient correction methods based on separation of dominant components in measured signals using singular value decomposition (SVD) have been presented for binned rotationally symmetric data (Haldrup, 2014 ▶) and for a general intensity field of a megapixel detector (van Driel *et al.*, 2015 ▶).

In the following we describe a simple formalism for a general complex spatially dependent nonlinear detector response. We show that under the boundary of a similar spatial signal distribution the problem becomes solvable and the nonlinear behavior can be approximated by that of individual pixels for which calibration curves can be experimentally determined. After deriving a nonlinear correction formula for each individual pixel in a most general form, we show a specific way of parametrization of the nonlinearity and application of the correction to data obtained using the CSPAD in the v1.2 generation as example. We show that the method is particularly suited for the challenging case of small changes within an intense diffuse signal and can reduce systematic measurement errors by around a factor of ten.

In our approach, the systematic behavior is isolated from random detector-induced effects and parametrized model-independently in relation to a calibration of known intensities in order to determine the detector gain field that may depend on the exposed pattern. As an example of application, pump/probe scattering data are treated by this method, where optically excited signal changes are tracked as a function of delay between the exciting optical laser pulse and the probe X-ray pulse.

A description of variables used during the mathematical derivation of a correction equation is provided in Table 1 ▶.

## 2. Signal composition   

The raw digital signal of one single image measured by a pixel array detector (PAD), an array 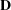 = 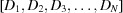, can be decomposed into a constant dark-current component 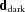, random photon exposure independent components which average to zero over many exposures 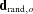 (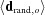 = 0), as well as a signal that is due to the exposure to photons 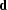:
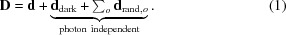
The random component can consist of different sources (accounted for by index *o*), such as individual pixel noise and components common to an entire detector segment. In the latter case, correction algorithms can be applied which analyse the intensity statistics of an entire image 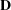 or a subset of that (*e.g.* physical detector segments), or use the signals from purposely unexposed pixels as noise reference. For an average sample of images 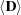, the randomly fluctuating signals average to zero by definition, *i.e.* the random detector behavior, if not accounted for, can be reduced by averaging over measurements of an identical signal. For this reason, the constant dark offset can be measured with high accuracy by averaging a large number of unexposed detector image acquisitions.

In this article, we focus on the photon-signal-dependent components summarized in 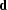, in which both the ideally linear response to physical signals but also potential nonlinear artifacts from, for example, pixel crosstalk are combined.

## 3. Example CSPAD dataset   

As an exemplary test case, we use a reference diffuse X-ray scattering dataset from a free-flowing 100 µm-thick liquid sheet jet of acetonitrile which was recorded for a range of different FEL X-ray intensities using the CSPAD. The data were recorded at the LCLS XPP instrument (Chollet *et al.*, 2015 ▶), where the FEL intensity can be controlled by inserting single-crystalline silicon attenuator blades of different thicknesses into the beam path before the sample. The data represent a high exposure of the CSPAD in low-gain mode with 5 ADU (analog-to-digital units) per 9.5 keV photon, *i.e.* the maximum shown in Fig. 1 ▶ represents approximately 2000 photons per pixel in the liquid peak and about 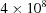 photons on the entire CSPAD.

The X-ray pulse intensity delivered to the sample was measured by a diode-based transmissive intensity monitor (Feng *et al.*, 2011 ▶). As the hereby measured intensities do not reflect changes of sample volume due to fluctuations in the liquid jet thickness, the total exposed intensity (after subtraction of the dark component) was used as the total intensity estimate (see remarks about this procedure in §7). After subtraction of the dark component, the X-ray patterns were sorted into equally spaced intensity intervals and averaged. The data in each interval were averaged in order to reduce the magnitude of random components in the digital data or the dependency to sample jet fluctuations.

A spatially dependent nonlinear detector response becomes visible when comparing this dataset of identical signal distribution measured at different total X-ray intensities (Fig. 1 ▶). While the signals from pixels at exemplary locations in the scattering pattern show predominantly a dependence on the total intensity, additional nonlinear deviations become visible after subtracting a fitted linear component. The nonlinear behavior clearly varies qualitatively and quantitatively at the different locations in the scattering pattern. Neighboring pixels which measure a similar signal of the smooth scattering pattern, however, behave qualitatively alike: when comparing average patterns measured at different intensities that were normalized to their total intensity, residual regions with amplitudes up to 10% of the actual signals are observed where pixel groups form elevated and reduced intensity regions [Figs. 2(*a*)–2(*c*) ▶]. These residuals, due to spatially dependent nonlinearity, increase with the difference of total X-ray intensity at which the compared patterns are measured. The intensity-feature shapes appear to correlate with the shape of the exposed signal pattern, which is a sign of pixel crosstalk. Additionally, features that are given from the individual detector tile appear overlaid with the intensity-dependent part of nonlinear components.

## 4. Decomposition and correction of the photon-dependent signal at similar exposures   

In order to contemplate the cross dependencies of the pixels we decompose the pixel array 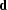 into its elements, the intensities per pixel 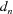 for the *N* pixels of the detector,
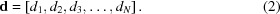
In a functional dependency view, the measured intensity on an individual pixel is a function of the physical photon exposure 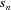 of that pixel but also of the measured intensities in all other pixels, when assuming a most generalized pixel crosstalk, 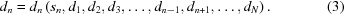
This is eventually equivalent to a dependence on the individual photon exposure experienced by each pixel or the total intensity field 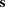 = 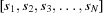:
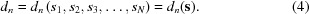
A full correction for the detector behavior would be possible with knowledge of the intensity dependence of each pixel to all possible intensity distributions on the detector. An experimental measurement of such a general calibration function, however, is highly unpractical. For a parametrization into *M* intensity intervals for each pixel, 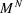 calibration measurements have to be taken which makes a general signal calibration for ≥14-bit megapixel detectors practically impossible. However, special assumptions about the detector and the photon signal 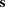 described by the total intensity field allow to some extent a quantitative comparison of the measurements.

For a specific constant photon density distribution 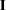 on the detector, the intensity dependence equation (4) can be reduced to a single non-constant scalar variable:
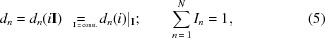
where *i* is a value that scales with the total photon intensity and 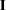 is normalized to unity. In this particular case the single-pixel response becomes a one-dimensional function dependent only on the scalar photon intensity *i* and a detector calibration dataset requires only 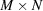 measured calibration values for the constant photon distribution under study. These can be acquired by measuring an identical signal on the entire detector as a function of the single external variable *i* which is proportional to the total intensity (*i.e.* the dataset described above can serve as a calibration dataset). We describe this general detector response for a given intensity distribution 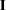 as a set of calibration functions 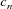 for each pixel of the detector:
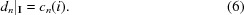
The functions 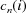 describe both the expected digital reading for each pixel given 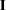 and the sensitivity to a change in intensity 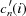 = 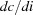. In order to quantitatively compare these *N* functions relative to each other, a calibration across all pixels is required, *i.e.* one point of exposure with a known signal on the calibration curves for each pixel. Here, we assume a calibration measurement at total intensity 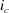 with a measured intensity 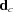 = 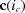 and the known calibration signal 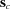, so that we can calculate a gain field 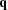 = 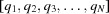 of ‘signal units per digital reading’ (*cf.* ‘gain map’ or ‘flatfield’ for a single intensity^1^) at that 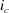 whose elements are
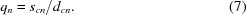
Note that we have chosen our correction around a point of reference at a general intensity value 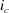 and not at the zero limit typical for nonlinearity correction of diodes. A reference at finite intensity values can often be the better choice because PAD detector artifacts like crosstalk may affect especially patterns of low exposure (Herrmann *et al.*, 2013 ▶). From that single reference point and assuming a constant signal distribution 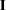 we can now trivially construct the linear corrected signal proportional to the total intensity scalar *i* as
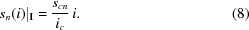
After we have defined calibration functions for each pixel under the assumption of a constant intensity distribution, we now consider a small intensity variation in a subset of pixels, *i.e.* the measured set of total intensity and individual pixel [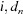 = 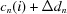] deviates from the ideally calibrated tuple [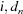 = 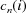]. For such small changes from the given 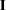, to first-order approximation, effects due to changes in pixel crosstalk would be very small. The intensity distribution, and therefore the distribution of charge load on the detector, is still very similar, and the calibration set given by 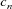 can be approximated as invariant. The total intensity parameter *i* can be regarded as defining a working point on the calibration curves 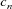, selecting a set of correction parameters. As the nonlinear behavior of 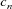 around a measured value *i* depends on the total load on the detector characterized by *i*, it does not determine the photon sensitivity of the individual pixels at that working point. The gradient 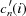, locally defined at *i*, represents the best estimate of the readout response 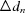 due to a change of external photon signal 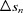. In general, 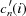 differs from the sensitivity at the calibration point 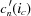 for which the gain 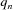 was determined by a reference dataset. For approximating the change in corrected signal 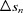 that is due to 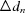, the gain therefore needs to be corrected by the ratio between the sensitivities at both points before being applied to the deviation 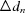 (see also schematic visualization in Fig. 3 ▶):
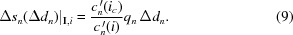
Under the assumption that the calibration data 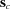 and *c* stay constant for small deviations from an intensity distribution 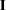, the corrected intensity can be calculated from the measured tuple 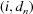 by
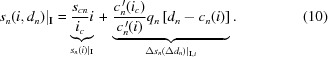
The result of this correction function has the same unit as the calibration dataset 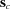. As the unit of the total intensity value *i* cancels out in this final correction function, any parameter that is proportional to the total intensity can be used in the correction process.

## 5. Parametrization of calibration and numerical data correction   

For practical application of the correction given by equation (10), an external calibration signal 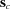 (see also discussion in §7) and a calibration dataset of the nonlinear detector defining 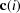 are required. An analytical differentiable parametrization of the *N* function elements in 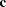 is preferable for efficient numerical evaluation. Additionally, an approximation by a fitted analytical function can help reduce undesired effects from statistical noise in the reference dataset. The best choice of such a function depends on the specific nonlinear effects and what physical models can be used to describe the behavior.

In the examples shown here, the calibration data are approximated by a Taylor expansion around the cross-calibration point 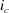,
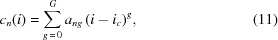
for which efficient least-squares fitting algorithms can find the parameters 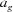 that match the calibration data best. The polynomial order can be truncated at degree *G* as required to characterize the features of the calibration data while minimizing the computational effort.

The dataset used in Fig. 1 ▶ was parametrized by this Taylor approximation for 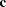 up to order 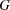 = 5 and 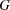 = 10 and the comparison of identical signals at different total X-ray intensities was repeated after the data had been corrected by equation (10) using 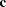 given by equation (11) (Fig. 2 ▶, second and third row). The systematic distortions could be significantly reduced, and the overall remaining residuals are reduced by a factor 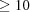. The degree *G* of correction clearly influences the quality of correction. For our example dataset, a five-order correction approximation of 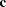 does not provide enough flexibility to represent the calibration dataset with sufficient detail.

For the polynomial approximation chosen here, it is straightforward to form the derivative 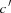, required in equation (10), and the gain at the calibration intensity 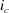 is given by the first-order parameters 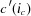 = 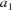. For practical numerical correction, the parameter 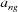, an 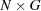-element dataset, can be kept in computer memory, and the correction reduced to a series of simple array operations which are ‘embarrassingly parallel’ to be run efficiently in numerical environments of high-level programming languages and/or on multiprocessor systems.^2^

As an example of realistic application, the described nonlinear intensity corrections were applied to data from a pump/probe X-ray diffuse scattering experiment with signal changes below 1%. The diffuse scattering from a 100 µm-thick jet of water was measured after exciting bending and stretching modes of the water molecules directly through a ∼75 fs short pulse of 1950 nm light. The non-corrected and corrected images were azimuthally averaged and binned into radial profiles of units of the absolute wavevector transfer 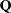. Difference profiles between excited and unexcited water were calculated and sorted for laser-to-X-ray time delay Δ*t* as measured by a timing diagnostics (Harmand *et al.*, 2013 ▶; Fig. 4 ▶). Each time delay bin contains an average of 50 difference scattering images resulting in ∼30 fs bin sizes for the presented dataset. After the pump/probe delay Δ*t* = 0, when the two pulses overlap in time, a difference signal characteristic for heated water (Cammarata *et al.*, 2006 ▶; Kjær *et al.*, 2013 ▶) grows in.

In the uncorrected case, strong fluctuations of similar magnitude as the physical light-induced signal changes dominate the extracted signal, even though the shown data represent averages of hundreds of pixels and multiple images. The average is therefore insufficient to accurately determine the 0.1% signal changes of interest. After correction, the fluctuations in direction of the pump/probe time delay 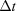 are reduced to below 0.1% of the total signal and the experimental information can more readily be extracted. In this example the signal shape fluctuations were dominated by intensity-dependent effects.

## 6. General application   

The method described in §4 and §5, in essence, finds the correlations of each pixel in a pixel detector with respect to a single parameter describing a physical property of one X-ray pulse, by averaging all acquired data in fine intervals of that parameter and thereby reducing the influence of all other fluctuation effects. Subsequently, a linear signal is calculated from the parameter, and deviations from the calibrated dependence are weighted according to the corresponding selected working point.

Instead of regarding deviations from an expected dependence, the parameter calibration can also be used to remove the correlation, again under the limit of similar exposure patterns. This can be useful for all those parameters which fluctuate, can be measured by a scalar signal and induce distortions image of the area detector. Removal of distortions caused by parameter *p* can be described by
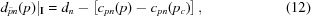
where 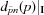 represents a ‘corrected’ digital reading without dependence on the parameter *p*, 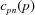 denotes calibration functions of the read signal as a function of parameter *p*, and 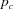 is a chosen calibration center.

FEL-related examples for parasitic signal distortions due to an external parameter *p* are the fluctuating photon energy and beam pointing parameters which can be measured by FEL diagnostics. In the example case of liquid scattering the fluctuating photon energy would change the wavevector transfer 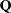 probed by an area detector which leads to small radial motions of the scattering features (van Driel *et al.*, 2015 ▶). As the photon energy can be measured for each pulse, either directly by a transmissive spectrometer (Zhu *et al.*, 2012 ▶) or indirectly by the energy of the FEL electron bunch, a calibration dataset can be sorted and averaged for that photon energy parameter and a calibration 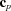 can be formed where *p* in equation (12) represents the photon energy. As a calibration parameter value 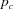 the average photon energy would be a well suited choice for the nominal value, and the images would be corrected to match the signal at this 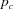. Also for other FEL-specific fluctuations related to beam pointing or temporal properties of the pulses, a parametrized correction as described in this article can be envisioned.

## 7. Practical considerations   

The procedure described here corrects complex nonlinear signal distortions model-independently through signal-dependent calibration. The nonlinearity correction relies on a representative calibration dataset of a constant signal measured at different intensities that span over the intensity distribution in the real measurement [defining 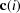]. In the case of typical FEL intensity fluctuations, a measurement over a large number of X-ray pulses along with the data would typically serve that purpose, as the intensity fluctuations between this dataset and the actual experiment would be comparable. A larger intensity range can be covered by purposely attenuating the incoming X-ray pulse intensity as described in §3. Such a dataset can directly be used to determine the calibration field 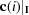 for the measured *i* range, *e.g.* by polynomial approximation as given in equation (11). In the case of additional fluctuations like common mode detector noise, sample volume fluctuations or similar, the effect of those parameters on the nonlinearity calibration dataset can be reduced by averaging the data of similar intensity into bins (performed in the example dataset shown in Figs. 1 ▶ and 2 ▶).

The quality of the nonlinearity corrections depends on the quality of the calibration dataset. But even data at lower statistics can lead to a significant reduction of nonlinearity-induced fluctuations. In case a dedicated calibration dataset has not been recorded, suitable reference measurements can often be identified within the recorded data. In the case of pump/probe measurements, a set of unexcited time-delays or reference images are usually acquired during the experiment. Such data are well suited for corrections as long as the intensity distribution and effective fluctuations are representative of those data to be corrected.

In the examples presented here, the CSPAD in the v1.2 generation was corrected at high intensity levels. The systematic nonlinear effects had been significantly improved compared with the v1.0 generation. As the crosstalk effects have been shown to affect also very low measured intensities with few photons per pixel (Herrmann *et al.*, 2014 ▶), the correction procedures can improve all intensity ranges at which the CSPAD is used. The newer v1.5 and the presently active v1.6 CSPAD generation represent another substantial step of reduction of crosstalk effects. Nevertheless, we have gained significant improvements of pump/probe data fluctuations by correcting for intensity-dependent nonlinearity for those newest generations. The large fluctuations of pulse intensities typical for FELs cause a high sensitivity to nonlinear effects. It should be noted that very small crosstalk effects can be expected at other types of integrating area detectors whose effects turn out to be less obvious when using very stable light sources like synchrotrons.

Other experimental techniques, not tested in this article, often regard single-image differences from a well established average of all images, and a correction of nonlinear detector response described here might reduce systematic detector-induced errors. In such cases the binning technique described in this article can help to generate a calibration dataset with reduced dependence on the individual signal changes.

In macromolecular crystallography, a major field of application of FEL radiation, artifacts of structure factor determination through detector-induced spatial and intensity-dependent signal contributions can be typically reduced by relating the Bragg reflection intensities to the background signal measured in their vicinity. Strong diffuse background signals due to amorphous liquid, however, can represent the major signal measured by the area detector, and thereby cause nonlinear artifacts in the measurement of Bragg reflections through pixel crosstalk. The correction concepts described here could help to reduce such effects because the small Bragg reflections represent a comparably small deviation from a constant intensity distribution, if the background is strong. Quantification of the background signal as well as measurement of a calibration dataset based on the background signal, independently of the strong Bragg reflections, can require advanced numerical methods like spatial frequency filtering. Correction of such data has not been tested. It should be noted that the systematic errors due to nonlinearity we present here in the CSPAD examples do not exceed a few percent of the signal and might be outweighed by other systematic uncertainties in a crystallography experiment.

As derived from equation (10), requirements for the total intensity parameter *i* are relaxed to a parameter which is proportional to the intensity. Nevertheless, a good measure of the total intensity can be difficult to obtain in practice, for example due to additional nonlinearity of intensity monitors or fluctuations in the sample as in the case for a liquid jet. In the presented example, the total intensity measured by the CSPAD detector was used as a reference intensity monitor. This procedure appeared justified by cross-correlation with an intensity monitor which suggested that the nonlinear effects of the individual pixels appear reduced when averaging over a large ensemble of pixels at different intensities. Depending on the level of nonlinearity and the exposure level of the detector, such a procedure might be inappropriate and return a nonlinear signal itself, and other means for an intensity monitor have to be identified.

The practical relative or even absolute gain calibration across all pixels using a single reference image 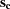 can represent a challenge, depending on the detector behavior. In the case of negligible pixel crosstalk at the low exposure limit, the zero approximation (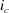 = 0) can serve as a good and most general reference point, which would be independent of the regarded intensity distribution. In this case a low-exposure flatfield measurement of a large number of pulses can serve as calibration and the important gain calibration 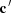
(*i* = 0) can be determined by extrapolation of 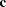 to *i* = 0. In case the calibration curves cannot be easily parametrized at the low exposure limit, for example due to discontinuous behavior through crosstalk [like in the case of the CSPAD, *cf.* Herrmann *et al.* (2014 ▶)], the actual intensity distribution might be required to serve as intensity calibration. In many cases the measured signal distribution (*e.g.* a liquid scattering pattern) can be or has been measured on a highly linear detector at a synchrotron source and can be used to calibrate intensities. For the direct comparison of such a reference, parameters dependent on the individual setup geometry (*e.g.* detector efficiency, scattering angles, X-ray polarization) have to be taken into account. Even without an intensity calibration dataset, the nonlinear corrections can be applied and the gain properties are determined by 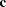
(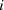 = 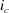).

The method presented here independently corrects any array of individual elements with respect to an intensity variable valid for all those elements. Its field of application is therefore more general compared with the approach on independent components which gains sensitivity with the dimensionality of the detector array (van Driel *et al.*, 2015 ▶). As the variable of fluctuation is required to be known and the dependence of the calibration to other variables can be reduced by averaging into intensity bins, correction of lower dimension data does not reduce its efficiency and validity. It can therefore also be applied to smaller subsets of pixels with applied masks or to reduced data where one element of the array includes information from multiple pixels. In those cases the numerical effort can therefore be reduced. The methods can be combined in cases where the origin of signal fluctuations is not unknown. Hence, the SVD-based approach would help to identify parameters that dominate the fluctuations, like the total intensity or other physical X-ray beam parameters (see §6). Thereafter, the calibration steps presented here would be applied in order to determine the nonlinear dependence of each of those identified parameters and reduce either dependencies or a nonlinear detector response.

## Supplementary Material

Example high-level code written in Python and MATLAB.. DOI: 10.1107/S1600577515005536/ig5029sup1.zip

## Figures and Tables

**Figure 1:**
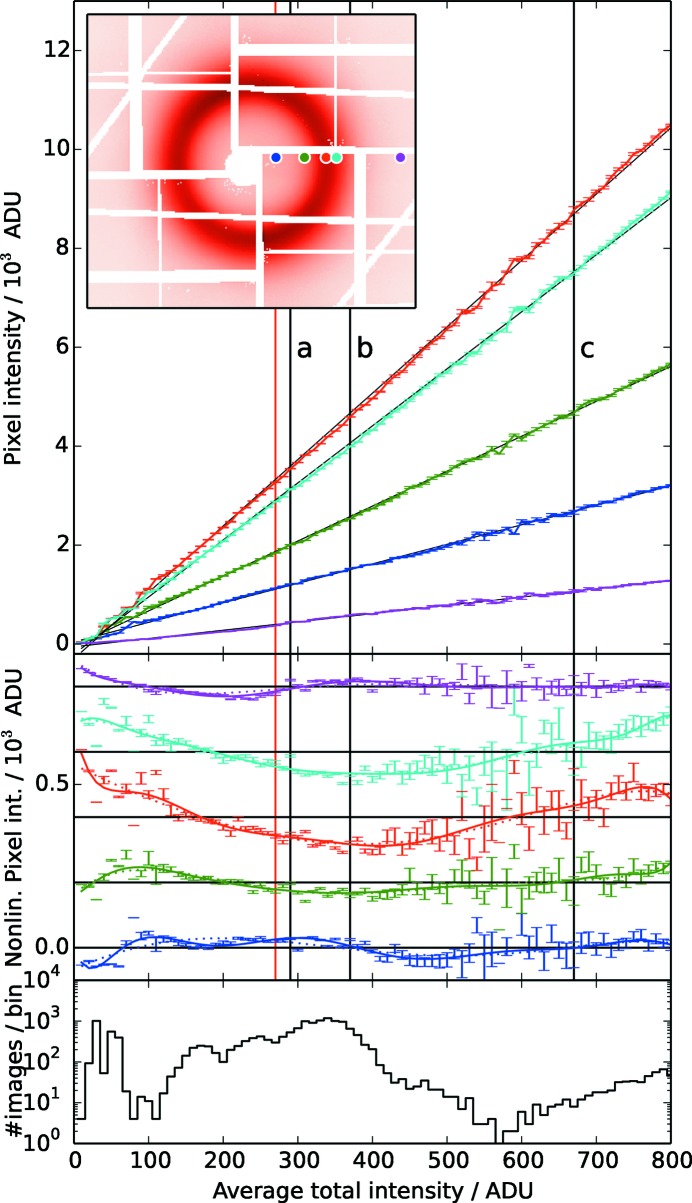
Top panel: total intensity dependence of five CSPAD pixels at characteristic positions in a diffuse scattering intensity distribution (indicated by round color symbols in the inset). The intensity units (ordinate), in analog digital units (ADU), represent the pixel-dependent parameter 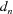 used in the mathematical descriptions. The data from multiple acquired images were averaged into bins of the total intensity (abscissa) given in units of the mean ADU intensity over the entire CSPAD pattern represented by *i* in the text [see also discussion after equation (10)]. The thin solid black lines represent calibration fits as described in §5. The middle panel shows the nonlinear residuals after subtracting this first-order polynomial fit. The residuals from each pixel have been offset for visibility and error bars corresponding to the standard deviation in each bin have been added. The varying statistics in each intensity interval effect the point-to-point variation; the bottom panel shows the number of images in each bin. The red (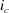) and black vertical lines (*a*, *b*, *c*) are example intensities used in Fig. 2 ▶.

**Figure 2:**
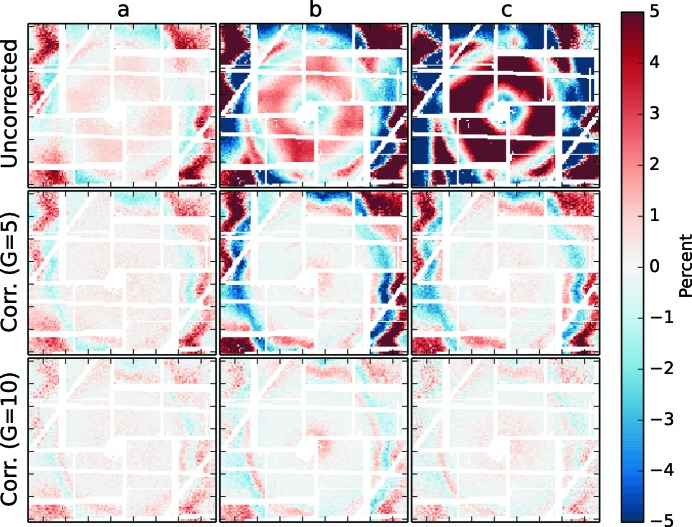
Comparison of the shape of acetonitrile diffuse scattering patterns recorded at different incoming X-ray intensities *i* (indicated *a*, *b* and *c* in Fig. 1) with respect to a reference intensity (*cf.* 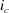 in the text, indicated by a red vertical line in Fig. 1). After the raw intensity patterns were normalized to their total intensity, their relative changes to the normalized pattern at the reference intensity are shown in color contrast as a percentage [*a*, *b*, *c* as columns, first row, *cf.* 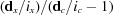 where *x* represents *a*, *b* and *c*]. The same comparison was repeated after correcting the patterns with equation (10) and parametrization with equation (11) up to to orders five and ten (maximum order *G*; middle and bottom row).

**Figure 3:**
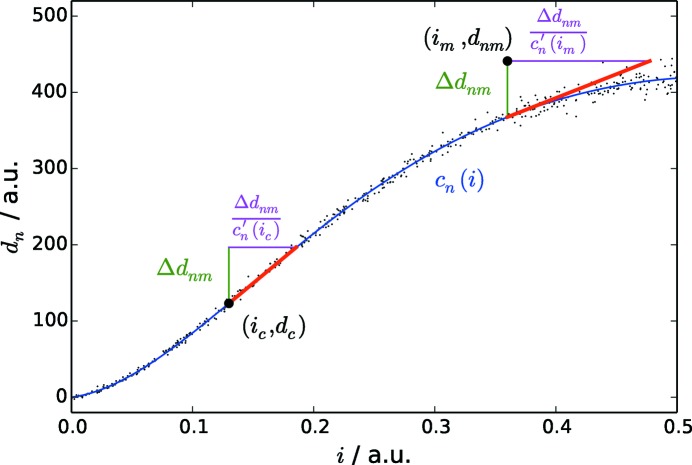
Schematic example of the correction of gain for a single pixel. Artificial example data of exaggerated nonlinearty show the dependence of a single pixel reading 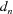 as a function of the total intensity *i* at a constant intensity distribution on the entire detector (small black dots). The data have been parametrized to form a calibration curve 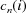 (blue line). An example data tuple 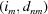 shows a deviation 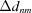 from the expected calibrated value 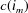. This deviation is subject to a different intensity dependence (gain) than at the the intensity 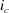 at which a quantitative calibration dataset was taken. This deviation in detector response can be accounted for by the ratio 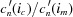.

**Figure 4:**
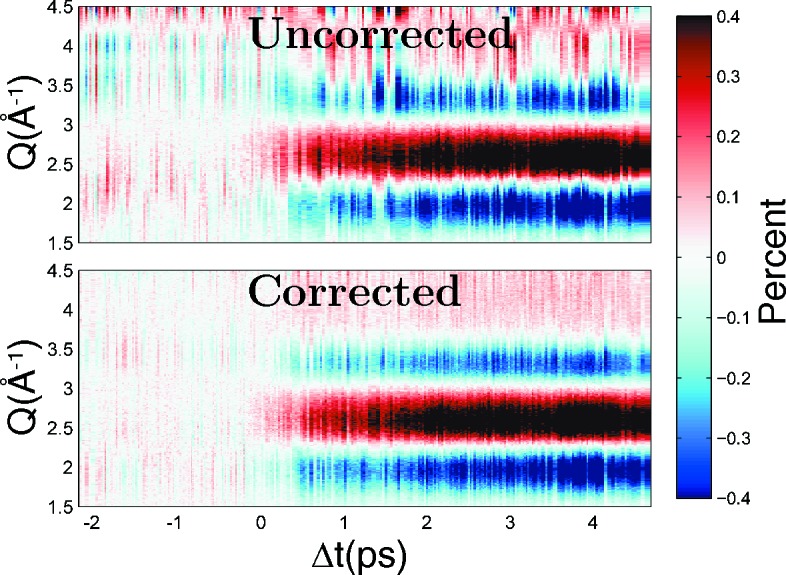
Example application of the nonlinear intensity correction presented in the article. The correction was applied to a typical X-ray diffuse scattering pump/probe experiment measuring the scattering response of prompt heating of water pumped with a ∼75 fs, 1950 nm laser pulse. The individual scattering images have been corrected, subtracted from unexcited reference images and azimuthally integrated to form difference scattering curves. The color contrast shows the relative change in scattered intensity for different scattering vectors as a function of time-delay between pump and probe pulse. After correction (lower panel) the signal fluctuations between time-bins are significantly reduced.

**Table 1:** Description of important variable names used in the article; when applicable, the element names of array variables which describe parameters for each pixel of an area detector are listed in the ‘Elements’ column

Symbol	Type	Elements	Description
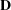	Array	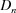	Raw digital area detector data
*N*	Integer		Total number of pixels in detector
*n*	Index		Pixel index 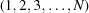
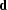	Array	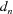	Photon-dependent digital data contribution
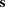	Array	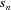	Photon signal on each pixel
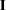	Array	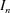	Given constant intensity distribution, normalized to 1
*i*	Scalar		Total detector intensity
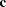	Array	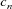	*i* dependence of 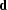 at constant intensity distribution, used as calibration dataset
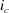	Scalar		*i* reference point for calibration across pixels
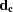	Array	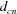	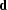 at 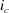
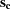	Array	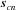	The expected signal expected at 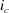 , used to calibrate the absolute units.
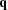	Array	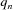	Gainmap
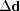	Array	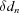	Deviation of the measured intensity from the calibration 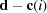
*G*	Integer		Maximum Taylor expansion order used for used approximation of 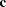
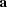	Matrix	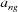	Polynomial parameters used to approximate 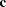 ; polynomial order *g* ranges from 1 to *G*
*p*	Scalar		General physical example parameter
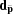	Array	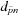	Detector signal reduced for dependency to parameter *p*
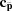	Array	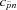	*p* dependence of 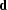 at constant intensity distribution

## Footnotes

1 Note that the definition of gain used here for flatfield correction is the reciprocal of the definition often used when describing the electrical signal gain of hardware.

2 An example high-level code written in the languages Python and MATLAB is available as supporting information to this article.
